# Visual attention to faces in children with autism spectrum disorder: are there sex differences?

**DOI:** 10.1186/s13229-019-0276-2

**Published:** 2019-06-28

**Authors:** Clare Harrop, Desiree Jones, Shuting Zheng, Sallie Nowell, Robert Schultz, Julia Parish-Morris

**Affiliations:** 1 University of North Carolina at Chapel Hill, Allied Health Sciences, Bondurant Hall, Chapel Hill, NC 27599 USA; 2 School of Behavioral and Brain Sciences, University of Texas–Dallas, Richardson, TX 75080 USA; 3 STAR Center for ASD and NDDs, University of California San Francisco, San Francisco, CA 94143 USA; 4 Frank Porter Graham Child Development Institute, University of North Carolina at Chapel Hill, Chapel Hill, NC 27599 USA; 5 Center for Autism Research, Children’s Hospital of Philadelphia, Roberts Center for Pediatric Research, Philadelphia, PA 19146 USA; 6 Perelman School of Medicine, University of Pennsylvania, Philadelphia, PA 19104 USA

**Keywords:** Autism spectrum disorder, Sex differences, Social cognition, Eye gaze, Social attention

## Abstract

**Background:**

The male bias in autism spectrum disorder (ASD) diagnoses is well documented. As a result, less is known about the female ASD phenotype. Recent research suggests that conclusions drawn from predominantly male samples may not accurately capture female behavior. In this study, we explore potential sex differences in attention to social stimuli, which is generally reported to be diminished in ASD. Population-based sex differences in attention to faces have been reported, such that typically developing (TD) females attend more to social stimuli (including faces) from infancy through adulthood than TD males. It is yet unknown whether population-based sex differences in the face domain are preserved in ASD.

**Methods:**

A dynamic, naturalistic infrared eye-tracking paradigm measured attention to social stimuli (faces) in 74 school-aged males and females with ASD (male *N* = 23; female *N* = 19) and without ASD (male *N* = 16; female *N* = 16). Two kinds of video stimuli were presented that varied in social content: *rich* social scenes (dyadic play between two children) and *lean* social scenes (parallel play by two children).

**Results:**

Results revealed a significant 3-way interaction between sex, diagnosis, and condition after controlling for chronological and mental age. ASD females attended more to faces than ASD males in the socially lean condition. This effect was not found in the typically developing (TD) group. ASD males attended less to faces regardless of social context; however, ASD females only attended significantly less to faces compared to TD females in the socially rich condition. TD males and ASD females did not differ in their attention to faces in either condition.

**Conclusions:**

This study has implications for how the field understands core social deficits in children with ASD, which should ideally be benchmarked against same-sex peers (male and female). Social attention in ASD females fell on a continuum—greater than their ASD male peers, but not as great as TD females. Overall, their social attention mirrored that of TD males. Improved understanding of the female social phenotype in ASD will enhance early screening and diagnostic efforts and will guide the development of sex-sensitive experimental paradigms and social interventions.

## Background

### Social attention in autism spectrum disorder

Reduced social attention is a core characteristic of autism spectrum disorder (ASD) that has been well characterized using infrared eye tracking [1]. Differences in social attention are evident by the second year of life and predict eventual ASD diagnosis [2, 3] as well as later social competence [4]. Studies using a variety of stimulus types have revealed social attention deficits in ASD [4–8], but there is evidence to suggest that some types of stimuli elicit larger group differences than others.

Eye tracking stimuli can be characterized on continuum of social complexity and ecological validity, ranging from faces presented in isolation or with competing non-social stimuli (e.g., [8, 9]), to faces embedded within static scenes or scrambled [10], to naturalistic social scenes depicting individuals interacting with one another [11, 12]. When faces are presented with competing non-social stimuli, children, and adolescents with ASD attend less to faces than matched peers [8, 9, 13]. This effect is particularly evident when the non-social stimuli overlap with common interests in ASD [8, 9, 13, 14].

Recognizing that faces are not presented in isolation in real-world situations, a handful of recent eye-tracking studies demonstrated that utilizing dynamic, ecologically valid scenes depicting complex social interactions elicits greater diagnostic group differences in social attention. Chawarska et al. [15] reported reduced attention to dyadic social stimuli (gaze and child-directed speech) by toddlers with ASD. This effect was not observed in the absence of these cues, suggesting a context-driven social attention deficit that was most pronounced during rich social scenes. Similarly, Speer et al. [12] reported the largest group effects when children with ASD viewed social-dynamic stimuli, with reduced attention to eye regions and increased attention to body areas relative to controls. Chevallier et al. [11] manipulated the nature of social stimuli, comparing static and dynamic stimuli that varied from socially lean (pictures of faces and objects) to socially rich (videos of children using a variety of nonverbal cues to interact together). Naturalistic social scenes elicited larger attentional differences than non-interactive static stimuli in children with and without ASD. Taken together, these studies suggest that social attention deficits are sensitive to contextual factors, particularly the social *richness* of a scene.

### Sex differences in social attention: typical development and ASD

While social orienting is assumed to reflect a core social challenge in ASD, several eye-tracking studies have failed to replicate the finding that individuals with autism always look less at faces than typical individuals [6, 16]. Two possible explanations could account for this discrepancy; first, stimuli may fail to capture the complexity of *real-world* social orienting and attention, and thus groups perform similarly. Second, researchers often fail to consider potential moderating effects of biological sex on attention to faces.

Males are four times more likely to be diagnosed with ASD than females [17], which is now understood to underestimate the true prevalence of ASD in women and girls [18]. Failure to identify ASD in females occurs in the context of a male-referenced conceptualization of the disorder, and the autism literature is filled with predominantly male samples. Consistent with this broader trend, the majority of eye-tracking studies include insufficient numbers of ASD females to assess potential sex differences in social attention. However, emerging literature suggests that the social experiences and behaviors of females with ASD differ from males in a variety of important ways that might suggest differentiated social attention. For example, females with ASD socialize differently than males [19], report more same-sex typical friendships [20, 21] and experience heightened social motivation [22]. A number of studies support the hypothesis that females with ASD are better at *social camouflaging* and use learned compensatory behaviors to mitigate their social challenges [19, 23–27]. Taken together, these studies suggest that males and females with ASD may demonstrate unique phenotypic profiles, and may necessitate a hunt for distinct biomarkers that identify males and females with ASD. For example, Bedford et al. [28] reported that a number of infant markers for subsequent autism are male-specific, and Kleberg et al. [29] reported a sexual dimorphism in infants at risk for autism, such that male infants at risk showed a more consistent pattern of reduced attention to eyes compared to controls, as compared to female peers.

The presence and nature of sex differences in social attention during infrared eye tracking is unclear, as few eye-tracking studies have examined sex differences in attention to faces in ASD and the majority of previous studies are underpowered to examine sex differences – even post hoc. For example, Riby et al. [9] included four females in a total sample of 28. Sasson and Touchstone [8] included just one female in their sample of 15 preschoolers with ASD. Speer and colleagues [12] did not include any females in their small sample of 12, and Chevallier et al. [11] included just four females in their large sample of 59 children with ASD. However, when sex has been adequately factored in the study design (through a more equal inclusion of ASD females), studies have begun to reveal sex-specific effects. Chawarska et al. [30] reported enhanced attention to social stimuli—including faces—in female infants at high risk for ASD. Harrop and colleagues [31] reported more normative patterns of social attention in ASD females when faces were paired with images of common circumscribed interests, suggesting that compared to ASD males, they were less influenced by non-social stimuli.

Evidence of quantitative sexual dimorphism in typical children’s attention to faces suggests the possibility of sex differences in ASD as well. In typical development, enhanced attention to faces has been reported in females relative to males across developmental periods; including neonates [32], infants [33, 34] and children, and adolescents [35] and across a range of paradigms, including eye tracking [33]. However, heightened attention to faces in females has not been reported by all [36], with some reporting enhanced identification by male infants and the presence of sex-specific face scanning strategies [37]. The extent to which potential sex differences are due to nature or nurture is widely debated, but researchers have argued that the sex imbalance in ASD (of which poor social orienting is a hallmark feature) indicates that basic sex differences in social attention are at least partially innate [32].

### Study aims and hypotheses

The aim of this study is to examine social attention patterns in school-aged females and males with ASD and typically developing (TD) controls when viewing scenes that are socially rich or socially lean, using a validated interactive visual exploration paradigm [11]. Based on previous clinical and behavioral literature, we expected that ASD females would exhibit increased attention to social stimuli (faces) compared to ASD males, in line with reports of higher social motivation in this group [22], more normative patterns of female attention using static eye-tracking paradigms [31] and sex differences observed in typical children [32, 34, 38].

## Methods

### Participants

Four participant groups were recruited to test the contributions of (a) ASD and (b) biological sex to social attention: (1) ASD males; (2) ASD females; (3) TD males; and (4) TD females. Three ASD participants (1 female; 2 males) did not complete the eye-tracking task due to behavioral or attention issues during the testing procedures, and 5 additional children completed the task but were not included in the final sample due to insufficient attention (less than 20% gaze; see “Preliminary Analyses” section), resulting in a final sample of 74 school-aged males and females with ASD (male *N* = 23; female *N* = 19) and without ASD (male *N* = 16; female *N* = 16; Table 1). All participants met the following inclusion criteria: between 6 and 10 years of age; absence of seizure disorder, acute medical or genetic condition; and absence of uncorrected visual impairments. Participant age range was selected in light of a meta-analysis suggesting that behavioral differences between females and males with ASD are most often detected after the age of six [39] and compensatory behaviors (reported in particular for ASD females—[23, 24, 26]; Tint et al. [40]) are less likely to be fully developed.

**Table 1:** Sample characteristics

	Males		Females		Diagnosis effect	Sex effect
	ASD (*n* = 23)	TD controls (*n* = 16)	ASD (*n* = 19)	TD controls (*n* = 16)		
Age (years)	9.53 (.84)	7.68 (1.47)	8.33 (1.56)	7.93 (1.53)	*F* = 12.61, *p* = .001	*F* = 2.24, *p* = .14
Mental age (years)	9.72 (2.31)	9.95 (4.20)	7.93 (2.85)	9.61 (2.02)	*F* = 1.95, *p* = .17	*F* = 2.43, *p* = .12
SCQ score	15.00 (6.19)	3.50 (2.58)	13.74 (5.19)	2.00 (2.92)	*F* = 109.81, *p* < .001	*F* = 1.55, *p* = .22
Basic attention	78% (23%)	88% (13%)	79% (18%)	93% (11%)	*F* = 8.61, *p* = .005	*F* = .62, *p* = .43

Mean (SD) unless otherwise noted

*SCQ* Social Communication Questionnaire

Participants with ASD were recruited via the University of North Carolina at Chapel Hill Autism Research Registry. Inclusion in the Autism Research Registry requires a clinical diagnosis of ASD from a licensed psychologist or psychiatrist with the majority of referrals to the registry stemming from regional diagnostic and treatment clinics using gold-standard diagnostic measures (such as the ADOS [41] and ADI-R [42]. TD children were recruited via an email sent to the University of North Carolina at Chapel Hill Child Development Research Registry, advertisements on social media and word of mouth. The “current symptoms” version of the Social Communication Questionnaire (SCQ; [42, 43]) was completed by parents during their study visit as a further screening tool in the ASD and TD groups (TD participants all scored below 15, which is the clinical cutoff for ASD). Importantly, male and female children with ASD did not differ in symptom levels (as measured by the SCQ), suggesting that any differences in social attention between ASD females and males cannot be explained by differences in autism symptoms.

To derive non-verbal, verbal, and spatial ability scores and age equivalents for each participant, we administered the Core Battery of the Differential Ability Scales (DAS-II; [44]). Due to the inherent difficulty of recruiting ASD females, and their tendency to fall within the lower functioning end of the spectrum, exclusions were not made based on IQ and functioning. The final ASD and TD groups did not differ on mental age (MA; Table 1), but the ASD group was chronologically older than the TD group (*F* = 12.61, *p* = .001) and had significantly higher SCQ scores (*F* = 109.81, *p* < .001). Overall, males and females did not differ significantly on chronological age (CA) or MA (Table 1), but ASD males were older than all other groups (all *p* > .01). TD females, TD males, and ASD females did not differ in CA. Based on reports that ASD females tend to fall within the lower functioning end of the spectrum, we analyzed the difference between ASD males and females for MA. ASD males had significantly higher MA than ASD females (*t =* 2.25, *p =* .03), but a null model predicting gaze to faces in the overall sample did not find a significant effect of MA, and thus MA was not included as a covariate in subsequent models. A null model revealed greater gaze to faces by younger participants; to account for potential effects of CA on gaze behavior, this variable (centered) was included in all analytic models. Upon testing, all results remained significant with and without MA and CA as covariates.

### Eye tracking stimuli and dependent variable

**Fig. 1:**
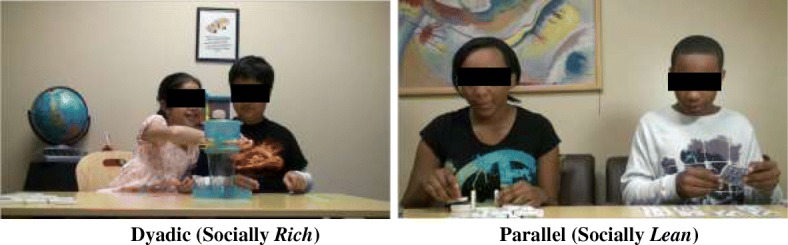
Representative stimuli from interactive visual exploration task

Given that the goal of this study was to assess social attention, the primary dependent variable was participant gaze to faces relative to overall gaze (as an index of social attention or preference). Stimuli and protocols for this study are described in detail in Chevallier et al. [11] and Parish-Morris et al. [45]; participants in the current study are non-overlapping. Subjects viewed 22 15.5-s video clips of sibling pairs engaged in parallel play (socially lean; not interacting with each other) or dyadic play (socially rich; interacting with one another). Each naturalistic scene consisted of child actors playing with toys either on the floor or at a table, with various objects in the background (Fig. 1). Children represented a range of ethnicities. Each set of siblings/scenes (including objects) appeared once in the socially rich condition, and once in the socially lean condition. Thus, low-level visual salience was controlled through identical faces/scenes in each condition.

Stimuli were shown on a screen with pixel (p) resolution 1920p × 1080p. Dynamic areas of interests (AOIs) were drawn onto each clip using Tobii studio. Face AOIs were ovals approximately 340p wide (~ 9 cm) and 440p tall (~ 11.64 cm), depending on individual child actors, which translates to visual angles of ~ 8.58° by ~ 11.08° at 60 cm viewing distance. Face AOIs were drawn slightly outside the stimulus bounds to account for possible drift due to slouching and other participant movements (e.g., ovals captured the actor’s face, part of her/his neck, and part of her/his hairline). Key frames created using Tobii Studio were used to adjust oval size and location as actors moved in 3D space (i.e., ovals became larger as actors moved closer to the camera and smaller as they receded), with dynamic interpolation between key frames. Face AOIs were grouped within each scene and condition for analysis. Fixation durations for each AOI group were summed across all scenes within each condition, to create total fixation duration variables for each AOI type (in seconds). An AOI that covered the entire screen for each trial was also created, in order to measure overall visual attention (fullscreen).

### Eye tracking parameters

Gaze data was exported from Tobii Studio using a filter based on the velocity-threshold identification (I-VT) fixation classification algorithm [46]. Fixation parameters were as follows: gap fill-in using linear interpolation was enabled, with a maximum gap length of 75 ms. An average of the right and left eyes was used to calculate fixation. Noise reduction was disabled, and the velocity calculator was set at 20 ms. Adjacent fixations were merged, with the maximum time between merged fixations set to 75 ms and the maximum angle between merged fixations set to 0.5°. Merging fixations close in time and proximity prevents longer fixations from being separated into shorter fixations because of data loss or noise. Fixations shorter than 30 ms that did not meet criteria for merging were discarded.

### Statistical approach

Analyses were completed in R [47]. Linear models (LM) or linear mixed models (LMM) assessed simple between-group (ASD/TD, male/female) effects on basic attention (see “Preliminary Analyses”, below). Random effects of participant ID (intercept) were included to account for repeated measures (e.g., comparing gaze in dyadic vs. parallel conditions). Cohen’s *d* is reported as a measure of effect size for linear models [48]. Following Cohen [48], *d* values between 0.20 and 0.50 reflect a small effect, between .50 and .80 a medium effect, and > 0.80 a large effect [6]. To account for individual differences in basic attention to the task (see “Preliminary Analyses”, below), raw gaze to faces was analyzed using generalized linear mixed-effects models (GLMM). In GLMM, gaze to the face was coded as a “hit,” while gaze to the rest of the screen (within condition) is coded as a “miss.” This solution allowed us to examine gaze to face AOIs relative to each individual’s total attention to the screen (rounded to the nearest second), answering the question of relative gaze distribution (preference) while controlling for basic differences in attention. GLMM were fit using maximum likelihood with a logit link, and reported using the *z*-statistic (similar to the *t* statistic from continuous linear models). Sex (female = 0, male = 1), diagnosis (TD = 0, ASD = 1), and condition [socially rich (dyadic) = 0, socially lean (parallel) = 1] were coded binomially. Odds ratios (OR) and 95% confidence intervals are reported for primary dependent variables and interactions. To test whether the distribution of residuals from our primary model violated normality assumptions, we used DHARMa [49]. DHARMa creates 250 new synthetic datasets to simulate the fitted model, calculates the cumulative distribution of simulated values for each observed value, and returns the quantile value that corresponds to the observed value. Using this method, we found that the residuals generated by our primary analysis did not violate normality assumptions (Kolmogorov-Smirnov *p* = .945; DHARMa nonparametric dispersion test, *p* = 0.496; DHARMa outlier test based on exact binomial test, *p* = .892).

### Preliminary analyses

#### Basic attention

Seventy-nine children completed the eye-tracking procedures. Five children were excluded from the analysis because they attended to the task less than 20% of the time (4 ASD, 1 TD). This parameter was set based on previous eye tracking studies (e.g., [31]). Based on prior research showing visual attention differences in ASD [50], we conducted further preliminary analyses to assess whether total fixation duration to the full screen differed by diagnosis and sex, both overall and by condition, after controlling for chronological age. Overall attention (raw gaze to the full screen) differed significantly by diagnosis (*t* = 3.14, *p* = .002; TD = 88%; ASD = 76%), but there was no significant interaction between diagnosis and condition. The TD group looked longer at the full screen than the ASD group in the socially rich condition (TD 88%, ASD 75%; *t* = 3.08, *p* = .003) and the socially lean condition (TD 88%, ASD 77%; *t* = 3.09, *p* = .003). To account for this difference, GLMM were used to assess relative gaze in our primary analysis (see “Statistical Approach” section). Overall attention to the task did not differ by sex (collapsed across diagnostic groups), and the effect of sex did not differ within condition. There was no significant interaction between sex and condition.

## Results

### Overall test

**Fig. 2:**
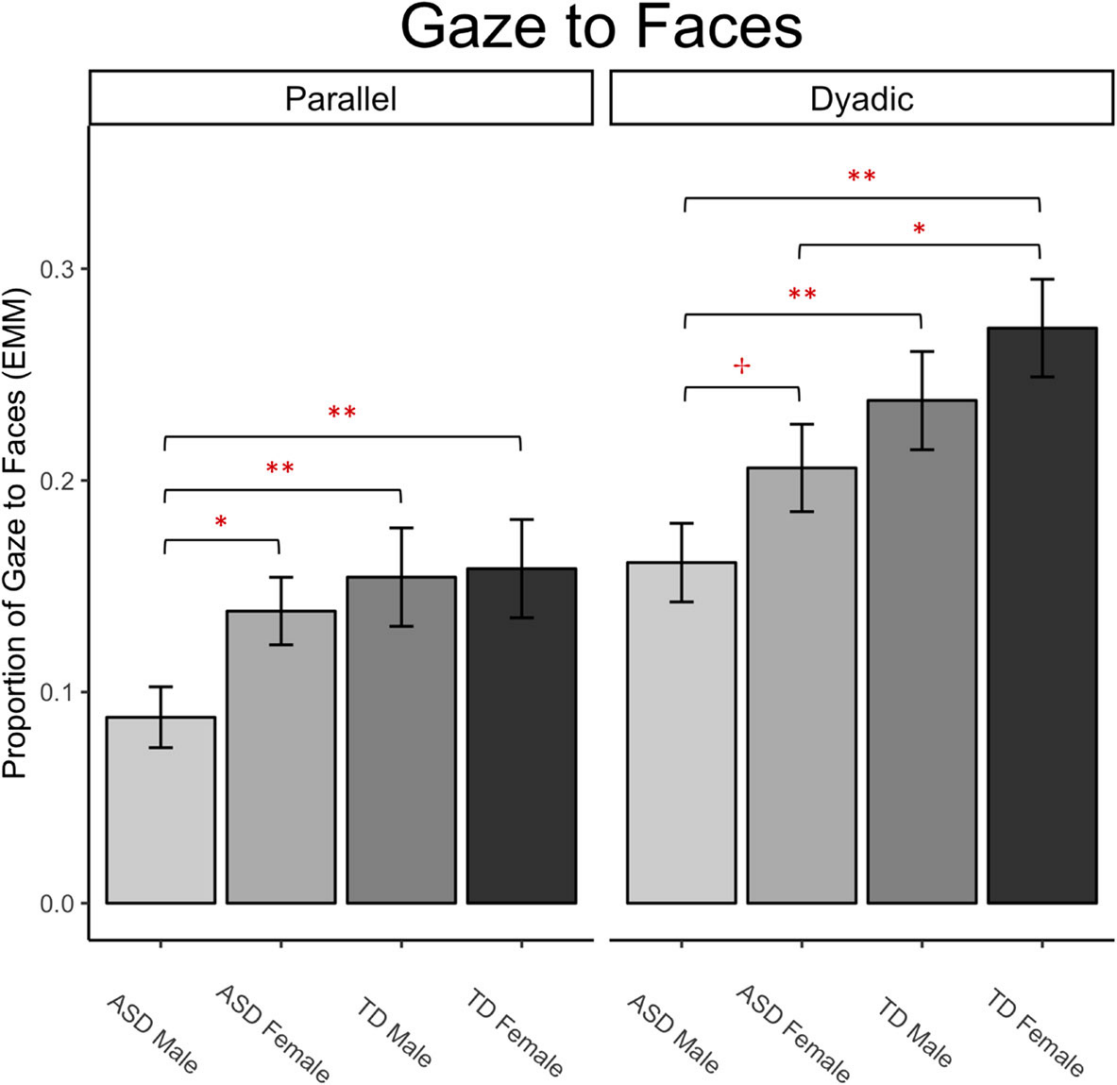
Estimated marginal mean gaze to faces (proportion of total fixation duration to faces relative to total looking time within each condition separately) after controlling for chronological age in a linear model. Notes: **p < .01, *p < .05, ✢*p* < .10. Pairs without significance symbols did not differ significantly from one another

An omnibus GLMM predicting gaze to faces revealed a significant 3-way interaction between sex, diagnosis, and condition (estimate = − .34, *z* = − 2.31, *p* = .02, OR.71, 95% CI .53–.95), after controlling for chronological age. This interaction suggests that face gaze (social attention) is different in boys vs. girls, ASD vs. TD participants, and in socially lean (parallel play) vs. socially rich (dyadic play) contexts. To assess the directionality of these results, follow-up GLMM models controlling for chronological age examined diagnostic group differences and sex differences across the two conditions. For a visual summary of the significant pairwise findings described below, see Fig. 2.

### Diagnostic group differences

Both male and female subgroups demonstrated diagnostic group differences in gaze to faces. In females, there was a significant diagnosis × condition interaction on gaze to faces (*z* = 2.28, *p* = .02; OR 1.25, 95% CI 1.03–1.52), driven by significantly less attention to faces by ASD females as compared to TD females in the dyadic condition (*z* = − 2.07, *p* = .04; OR .69, 95% CI .49–.98), and no significant diagnostic group difference in looking to faces during the parallel condition (*z* = − .72, *p* = .47; OR .69, 95% CI .87, 95% CI .58–1.28). There was no diagnosis × condition interaction in males (*z* = − 1.04, *p* = .30; OR .89, 95% CI .72–1.10). Separated by condition, additional tests revealed that ASD boys looked significantly less than TD boys at faces in the dyadic condition (*z* = − 2.88, *p* = .004; OR .51, 95% CI .32–.80), and in the parallel condition (*z* = − 2.72, *p* = .007; OR .39, 95% CI .19–.77), suggesting that ASD boys always looked less at faces than TD boys, regardless of social context.

### Sex differences

Significant sex differences in face gaze were evident in the ASD group, but not the TD group. In the ASD group, there was a significant sex × condition interaction on gaze to faces (*z* = − 2.16, *p* = .03; OR .79, 95% CI .64–.98), which was driven by significantly reduced gaze to faces by ASD males as compared to females during the parallel play condition (*z* = − 2.46, *p* = .01; OR .53, 95% CI .32–.88) but not the dyadic condition (*z* = − 1.74, *p* = .08; OR .70, 95% CI .46–1.05). There was no significant sex × condition interaction in the TD group (Fig. 2), nor any significant main effects of sex within each condition separately, suggesting that that TD male and TD female participants looked at faces in similar ways across the parallel and dyadic conditions.

Across diagnoses and sexes, our pattern of results reveals increased attention to faces in TD and female participants relative to ASD and male participants, particularly in the dyadic play condition (Fig. 2). Of note, whereas the looking patterns of TD girls and ASD boys differed from one another in both conditions, with ASD boys gazing significantly less to faces than TD girls (parallel *z* = − 2.41, *p* = .003; OR .46, 95% CI .25–.87; dyadic *z* = − 3.29, *p* < .001; OR .46, 95% CI .29–.73), girls with ASD and TD boys displayed similar patterns of social attention across the task (parallel *z* = .58, *p* = .56; OR 1.13, 95% CI .75–1.70; dyadic *z* = .17, *p* = .87; OR 1.04, 95% CI .68–1.59). This is consistent with recent imaging research suggesting that girls with ASD are more similar to boys without ASD, as compared to either boys on the spectrum or typically developing girls [51, 52].

## Discussion

The goal of this study was to understand the contribution of biological sex to social attention in the context of ASD. Our data support prior research showing broad social attention impairments in ASD, with a handful of caveats. Namely, females with ASD demonstrated enhanced attention to faces relative to males with ASD when the social scene did not convey an interaction (socially lean scenes). This difference was observed only in socially lean scenes and did not extend to scenes conveying richer social information. ASD females attended relatively less to faces than TD females (and did not differ from ASD males) when the scene conveyed an interaction between the actors (socially rich), suggesting that normalized social attention may be context dependent. Our findings align with a recent study of social attention to static images in ASD, which showed that females produce normative gaze patterns [31] and support the hypothesis that ASD females may be able to compensate for some of their social difficulties through increased attention to faces, particularly during social situations that are not very demanding. These findings have implications for future research paradigms designed to measure social attention in ASD while also considering sex-specific phenotypic characteristics and preferences.

### Diagnostic group differences

Prior research showing that children with ASD look less at faces than typical children appears to be broadly accurate, but our study found distinct patterns of looking in males and females with ASD as compared to typical same-sex peers. Face gaze patterns of ASD males differed significantly from TD males across the board. In females, the effect of ASD was less evident in gaze patterns when watching children play in parallel (lean social context) and much stronger in rich social contexts. Thus, while social attention in females with ASD may be enhanced relative to males with ASD, deficits emerge when females observe rich social communicative interactions that carry greater social demand.

### Sex differences

ASD females looked relatively more at faces than their male counterparts, suggesting that ASD females display greater social attention than males. However, this effect only reached significance in the *socially lean* condition, suggesting that social attention in the richer dyadic condition was equally impaired in both sexes. Interestingly, despite mean differences, the gaze patterns of TD females and males did not differ significantly from one another. This could reflect a true lack of differences in patterns of social attention at this age and ability level or could be an issue of power since each subgroup only had 16 participants. Nonetheless, this result is striking given recent findings indicating sex differences in TD and ASD children using a less naturalistic paired-preference paradigm [31]. It is possible that due to the differences in how TD males and females socialize, our paradigm (though more naturalistic than previous studies) may not capture differences in face attention between TD males and females.

In this study, social attention in ASD females appeared to fall on a continuum—slightly better than ASD males, but worse than TD females (Fig. 2). When we directly compared gaze to faces in ASD females and TD males, no differences in social attention were evident. Thus, our results provide support for the notion that social processes in ASD females may be comparable to TD males, which is also suggested by the results of a recent imaging study [52]. This hypothesis clearly warrants further investigation. Interestingly, our findings did not reveal differences between TD males and females in attention to faces. TD females demonstrated the greatest social attention across conditions, but this did not differ significantly from TD males (although this may be due to low statistical power, see “Limitations and future directions” section). There is a surprising dearth of studies on face processing in TD children, despite the common wisdom that females are more attuned to faces across developmental periods. This is a promising future research direction, as it will set benchmarks for understanding atypical development in males and females.

### Implications

#### Re-evaluating prior research

Significant prior research on gaze to faces vs. objects, and social vs. non-social stimuli in ASD exists in the literature, but very few studies examined sex differences. In light of the results reported here and those of Harrop et al. [31], prior findings should be interpreted with greater nuance. In the event of unbalanced sex ratios and small samples, it is possible that sex differences within and between groups confounded the results. For example, relatively fewer females in the ASD group vs. the TD group may have pushed the ASD mean into the “significantly different” range; thus, variable effect sizes and reproducibility problems in prior research may be due to the unexamined influence of biological sex. Moving forward, considering sex as a biological variable is imperative, just as it has long been acknowledged that chronological age and MA are important potential factors to consider when studying social perception in ASD.

#### Greater face gaze in ASD females: innate difference or learned compensatory strategy?

The findings reported here suggest that social attention in ASD females is more intact than social attention in ASD males, and yet differs in crucial ways from social attention in TD females. There are at least two potential explanations for this finding: (1) there is a fundamental biological sex difference in ASD, such that social attention is enhanced in females relative to males, despite comparable social impairment (the SCQ scores of ASD males and ASD females did not differ significantly in this study), or (2) ASD females have learned to compensate for social difficulties by increasing their attention to faces, but struggle to apply this strategy in socially demanding contexts. Either explanation may partially account for observed sex ratio differences in ASD, particularly at the higher end of the spectrum, and a combination of these factors likely leads to the unique social attention patterns in ASD females reported here.

#### Implications for clinical assessment and intervention

The findings reported here have implications for assessing autism in females. Although females with ASD may attend to other people more than males with ASD (and thus appear more socially aware at first blush), it is critical to recognize *how* they are attending. The current study suggests that females with ASD may attend to social information at less-optimal times than typical females, and thus consistently miss the kinds of important information that can be conveyed during rich social interactions. In terms of intervention, the findings reported here suggest that while both males and females with ASD could benefit from interventions designed to enhance attention to the social aspects of a scene, the nature of these interventions might differ. Our results suggest that targeted interventions focused on attention to faces during social interaction may be especially beneficial for females with ASD. However, given reports of increased anxiety and stress associated with behavioral *camouflaging* [19, 23–27], it is important to recognize that *if* females with ASD are taught to compensate socially through increased attention to faces, this may have unintentional downstream effects. Thus, there may be a trade-off between the benefits of enhanced social attention and potential negative effects in other areas.

### Limitations and future directions

Despite the many strengths of this study, it also has some limitations. Our sample of females, while the largest reported in eye-tracking research to date, is still relatively small. Small samples may result in insufficient statistical power to detect small effects; this study therefore merits replication in a larger sample. A larger sample could clarify mean differences in looking to social stimuli, which we observed were always greater for females in both diagnostic groups, but did not reach statistical significance in the smaller TD group. This possibility—that typical sex differences in social attention are preserved in ASD—is consistent with emergent research showing that social features of autism demonstrate similar patterns of sex differences in TD and ASD groups across a variety of diagnostic tools [23]. A larger sample would also facilitate exploring visual attention to non-face AOIs (e.g., hands, background objects) by providing sufficient power to support the inclusion of AOI type as an additional factor in the analysis. Attention and time scale are additional limitations of the current study, since children with ASD attended less overall than TD children, and analyses were conducted on data collected over ~ 5 min of gaze behavior. It is possible that male and female children with autism show atypical attention to faces during different phases of social observation (e.g., during initial orienting or reorienting). Thus, a more fine-grained analysis of how social attention unfolds in girls and boys with ASD is a promising research direction.

Sex-sensitive paradigms represent a particularly important avenue for future research and development. The paradigm described here was not designed to assess sex differences; boy and girl actors were not equally represented (although they were balanced by condition) and toys were gender neutral, so it is impossible to determine whether gaze was influenced by actor sex or might be affected by the *gender* of the toys included in the scene. Future research using a novel paradigm is planned, which will allow us to assess effects of actor sex, manipulate the gender-normativity of toys, and consider the role of background/distractor objects in modulating visual attention. These variables have emerged as potential influencers in sex difference research in both TD [53–55] and ASD, with ASD males reporting a preference for same-sex peers [56] and differences in attention to *gendered* toys [57]. Further, a more naturalistic paradigm, such as a live social interaction [58] or videos of children engaged in group play and interactions on the playground might reveal patterns of visual attention that mirror differential social expectations placed on males and females [59].

The results reported here have implications for understanding other neurodevelopmental and genetic disorders, particularly those with sex differences in prevalence and/or phenotypic expression. For example, fragile X syndrome also has a sex imbalance in diagnosis weighted toward males with females expressing weaker symptoms [60, 61] and has characteristic social attention impairments that have been detected via eye-tracking [62, 63]. Future research comparing social attention across diagnostic boundaries and biological sex aligns with the National Institute of Mental Health’s Research Domain Criteria [64] and the pan-National Institutes of Health initiative to understand sex as a biological variable. Finally, 6 to 10-year-olds were selected as a suitable age range for this study, since sex differences have been reported in ASD [39] and learned compensatory behaviors may not yet be fully expressed. Future research on children in a younger age range would allow us to understand whether ASD female differences in social attention are learned strategies (emerging later in development), or true protective effects that are observed even in young females, potentially pre-diagnostically.

## Conclusions

Mounting cross-domain evidence suggests that ASD females are phenotypically distinct from ASD males—and may behave in ways that are overtly similar to TD males and females, at least in contexts with lower social demands. In this study, we found that social attention in ASD females was reduced compared to TD females, and fell on a continuum between ASD males and TD males. Understanding sex differences in ASD is critical for lowering the age of diagnosis in females, for designing the most effective evidence-based and individualized interventions that help children reach their full potential, and for making progress on understanding the biological basis of ASD.

## Abbreviations

AOI: Areas of interest
ASD: Autism spectrum disorder
CA: Chronological age
DAS: Differential abilities scales
LM: Linear models
MA: Mental age
SCQ: Social Communication Questionnaire
TD: Typically developing
